# Determining Microbial Niche Breadth in the Environment for Better Ecosystem Fate Predictions

**DOI:** 10.1128/mSystems.00080-19

**Published:** 2019-06-11

**Authors:** Emilie E. L. Muller

**Affiliations:** a Equipe Adaptations et Interactions Microbiennes dans l’Environnement, Université de Strasbourg, UMR 7156 CNRS Génétique Moléculaire, Génomique, Microbiologie, Strasbourg, France

**Keywords:** ecosystems biology, ecosystem services stability, generalist/specialist, integrated meta-omics, lifestyle strategies

## Abstract

Integrated omics applied to microbial communities offers a great opportunity to analyze the niche breadths (i.e., resource and condition ranges usable by a species) of constituent populations, ranging from generalists, with a broad niche breadth, to specialists, with a narrow one. In this context, extracellular metabolomics measurements describe resource spaces available to microbial populations; dedicated analyses of metagenomics data serve to describe the fundamental niches of constituent populations, and functional meta-omics becomes a proxy to characterize the realized niches of populations and their variations though time or space. Thus, the combination of environmental omics and its thorough interpretation allows us to directly describe niche breadths of constituent populations of a microbial community, precisely and *in situ*. This will greatly facilitate studies of the causes influencing ecosystem stability, resistance, and resilience, as well as generation of the necessary knowledge to model and predict the fate of any ecosystem in the current context of global change.

## Abstract (precis)

Integrated omics applied to microbial communities offers a great opportunity to analyze the niche breadths (i.e., resource and condition ranges usable by a species) of constituent populations, ranging from generalists, with a broad niche breadth, to specialists, with a narrow one. In this context, extracellular metabolomics measurements describe resource spaces available to microbial populations; dedicated analyses of metagenomics data serve to describe the fundamental niches of constituent populations, and functional meta-omics becomes a proxy to characterize the realized niches of populations and their variations though time or space.

## PERSPECTIVE

Microorganisms are ubiquitous. Without them, numerous crucial functions in global ecosystems would not be as they are. To further understand and model the impact of forces that influence key microbial functions, we need to discern which measurable features of microbial communities are predictors of the stability of an ecosystem service.

For this, classical approaches have involved inventories of species (or operational taxonomic units as their contemporary molecular equivalents) and computing corresponding diversity indices, with more diverse environments being supposed to be more stable. Strategies that use microbial diversity as a predictor of ecosystem services and function stability rely mainly on the “unified neutral theory of biodiversity” (1), which posits that distant taxa can be functionally equivalent. However, the conclusions derived from such analyses have been limited (2). Hence, being able to define whether some species are ecologically equivalent or not is of crucial relevance to the understanding of the role played by microbial biodiversity in ecosystem functioning, as well as for environmental monitoring and diagnostics.

To address this challenge, recent initiatives aim at generating catalogues of microbial biodiversity, within specific environments (e.g., the MiDAS Global Database for wastewater treatment systems [3]) or at the global scale (e.g., the Earth Microbiome Project [4]), as well as related genomic content. Nevertheless, clear relationships between community composition, genetic diversity, and ecosystem functions cannot reliably be inferred, due largely to the vast diversity of microbes, many of which are still unknown in natural environments, and to limitations in deriving microbial functions from taxonomical analysis because of extensive horizontal DNA transfer in the microbial world. Also, the current lack of comprehensive information on the roles and interactions of a given type of microorganism in its environment prevents streamlined model building to predict system fate in the face of perturbations.

Thus, additional parameters to (bio)diversity measurements (2) might be necessary to improve the predictability of current models. Ideally, such parameters should be precisely measurable and quantifiable *in situ*, as well as directly applicable to feed model building. However, they are not fully identified yet. Hence, an exciting prospect and an important step of environmental omics, especially in the context of ongoing global change, are to develop our understanding of the ecological basis behind microbial community structure and population sizes, in other words, of microbial diversity.

## WHY IS IT SO DIFFICULT TO DETERMINE THE ECOLOGICAL NICHE BREADTH OF AN ORGANISM?

Textbooks on ecology explain that both community structures and population sizes are governed by resource availability and usage. However, resource usage is itself dependent on the ecological niche breadths of constituent populations across the continuum, from specialists that use a narrow range of resources and conditions, to generalists that take advantage of a wider ecological space. However, even if the determinants of ecological niche breadth are still poorly understood, it is commonly recognized that different specific metabolic capacities, usually carried by specific microorganisms, are necessary to occupy distinct ecological niches. Yet, the features measured to distinguish generalists from specialists still appear mostly author dependent. Some scientists define specialist species as taxa observed in a restricted number of different habitats, while others see them as organisms carrying at least one rare function in the ecosystem. Although these features describe interesting ecological properties of populations/organisms and are not mutually exclusive of the concept of specialist, they should not be considered as interpretable criteria to distinguish populations/organisms. This is because these descriptors do not relate directly to the niche breadths of the populations. Alternatively, some scientists define physicochemical gradients allowing growth or factors limiting it. Others look at the diversity of usable food (prey, carbon sources). These discrepancies are due to the notorious difficulty of simultaneously measuring the “n-dimensions” of niche hypervolume that define the fundamental ecological niche of a given population (see, e.g., reference 5), as originally defined by the zoologist Hutchinson in the middle of the last century (6). Indeed, according to this definition, fundamental niches are the exhaustive inventory of resource ranges and conditions usable in the absence of environmental stress, competition, or predation. However, describing the realized niche breadth of a population, i.e., the part of the fundamental niche that is actually used in the presence of other species and in a particular environment, is also challenging since each individual can have a slightly different one (7, 8). This has represented a major hurdle for investigating the higher organisms that are the historical models in ecology. Today, however, integrated omics applied to microbial communities offers a great opportunity to tackle these issues because of the possibility of going beyond the organism level by generating omics data at the population level.

## DESCRIBING MICROBIAL ECOLOGICAL NICHES *IN SITU* USING INTEGRATED META-OMICS

Technological advances related to omics technologies and big data analyses are now allowing scientists to process space- and time-resolved molecular information generated at high throughput with increasing quality. Thus, integrating multiple levels of meta-omics information is now a practicable solution to describe both fundamental and realized population-level niche breadth, especially in the microbial realm.

**FIG 1:**
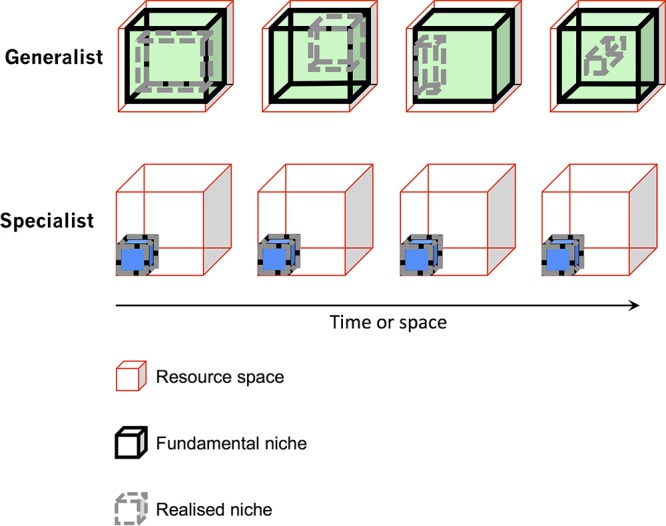
Integrated omics-driven *in situ* determination of ecological niche breadths of constituent populations of a microbial community. The resource space (in this example, a rather stable environment) can be characterized by extracellular metabolomics measurements coupled with physicochemical data. The fundamental niche of an organism living in this environment is described by its genomic complement, assumed here for clarity to be stable over time. The part of the fundamental niche actually used at a moment in space and time can be described by functional omics measurements (metatranscriptomics and/or metaproteomics) and is expected to be dynamic. A generalist species will occupy different fractions of its fundamental niche depending on biotic and abiotic conditions. In contrast, an organism with an extremely narrow niche breadth will always perform the only few functions encoded by its genome.

Specifically, while a combination of physicochemical parameters and extracellular metabolomics measurements describe resource space, metagenomics can be seen as a descriptor of fundamental niches by delineating the metabolic potential of each constituent population (based on analyses of metagenomically assembled genomes, or MAGs), with no restriction in the number of measured features. Further, functional meta-omics, such as metatranscriptomics and metaproteomics, is a proxy for the realized niche breadths of populations at each point in space and time (9, 10). Thus, the integration of all available environmental omics studies allows us to define both fundamental niche breadth and the difference between the fundamental and realized niches of a given population *in situ* (Fig. 1). Additionally, variations of realized niche breadths through time and space (i.e., metabolic flexibility [larger for generalists and smaller for specialists]) represent valuable information, available with detailed time and space resolutions of meta-omics data, to support these analyses and to understand biotic interactions at the ecosystem level.

## CHALLENGES MET, OTHERS STILL TO BE OVERCOME

The development and application of wet- and dry-lab procedures to extract, analyze, and integrate multiple levels of meta-omics information derived from environmental samples come with some challenges (11). First of all, the behavior of a generalist or a specialist microbial population needs to be validated through multiple time points and locations. Thus, a detailed procedure, robust to both the experimenter and the batch effect, is necessary for reproducibility of the obtained results. Second, because of the dynamic nature and extensive heterogeneity of mixed microbial communities, it is necessary to obtain biomolecules from an undivided sample and to avoid sample splitting (11). Curiously, protocols allowing a comprehensive biomolecular extraction for the simultaneous recovery of high-quality extracellular and intracellular metabolites, DNA, RNA, and proteins were not sought originally, despite their obvious value and advantages. Such protocols have now been available for some years (see, e.g., reference 12). Demonstrated to be automatable, they nevertheless require optimization for each new type of sample. Along the same lines, a robust and reproducible bioinformatics pipeline to integrate metagenomics and metatranscriptomic data is now also available (13), and further development will allow us to process metaproteomics data as well. Another challenge involves efficient computation of pairwise comparisons between the produced data/contigs/MAGs for each sample. Such “dereplication” makes it possible to obtain nonredundant population-level genomes, trackable over time and/or over samples (14).

Despite such recent progress, some questions still remain completely open. For example, how deep should functional omics measurements (metatranscriptomics and/or metaproteomics) be in order to comprehensively describe the realized niche of a population? In other words, can we determine the niche breadths of low-abundance organisms for which we may be unable to detect the full RNA or protein complement? This question is even more crucial for populations that are pivotal to ecosystem function despite only moderate abundances (i.e., keystone species). For the moment, only soft thresholds and manual curations are available to select which organisms can be confidently analyzed for their niche breadth and which should not be.

In addition, due to the high number of genes of unknown function in microbial genomes, counting the number of genes present or expressed currently represents the only realistic parameter to estimate fundamental or realized niche breadths, respectively. Thus, it is important to continuously support traditional and cutting-edge microbiological wet-lab approaches and to continue to develop databases for experimentally based gene annotation. Such a strategy will likely alleviate this identified limitation by bringing about a more extensive understanding of the functions encoded and expressed by microbial populations. We can even envisage obtaining predictions of the stability of encoded proteins as a function of pH, temperature, or oxygen concentration based on their sequence. This will give us extensive knowledge of the physicochemical range in which activities of interest are possible and, thus, a finer estimation of niche breadths. For now, however, this information is not yet “readable” in DNA sequences.

## BEYOND NICHE BREADTH: WHAT IS NEXT?

Determining the niche breadths of key members of a microbial community as outlined above will allow us to (i) test the link between community structures in terms of generalists/specialists with the stability and resilience of microbial communities faced with perturbations, and (ii) estimate the risks of collapse for microbially driven ecosystem services in a changing environment.

**FIG 2:**
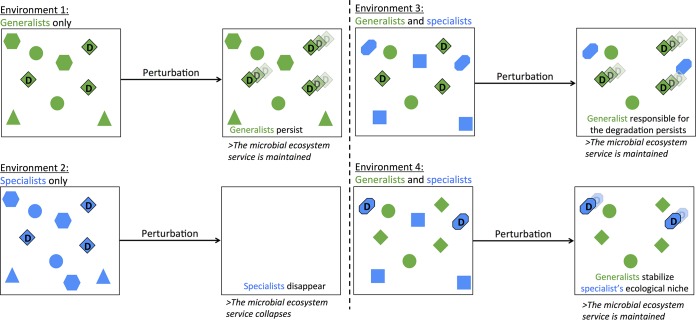
Potential effects of microbial niche breadth distribution on ecosystem service stability in the face of perturbations. We hypothesize that within endogenous microbial populations, generalists (green) with a large niche breadth and wide metabolic amplitude and specialists (blue) with a narrow niche breadth and restricted metabolic flexibility will affect the stability of ecosystem services (here, for example, the degradation of a pollutant) in different ways. Following perturbation, a community made up of only generalists (environment 1) will persist; the population(s) of the organism(s) responsible for this function (depicted with a “D” for degradation) might even expand (duplicated, shaded symbols) if the pollutant serves as a carbon or energy source, and ecosystem services will be maintained. For a community composed of only specialists (environment 2), the ecosystem service will disappear with the collapse of the population performing the degradation function. In more realistic environments, inhabited by both generalists and specialists, ecosystem services are predicted to be maintained if the related function is carried by generalists (environment 3), which is similar to the situation in environment 1. In environment 4, where the degradation function is carried by a specialist population, the studied ecosystem services may disappear following perturbation, or persist if associated generalists stabilize the ecological niche of the specialist, or take over the relevant function by horizontal gene transfer, leading to a situation similar to that in environment 1.

My current working hypothesis is that maintaining desirable microbial functions, e.g., functions associated with bioremediation, is linked to microbial community composition, structure, and dynamics, including the underlying balance between metabolic specialization and generalist lifestyle strategies. In other words, the key is not which organisms make up the microbiome (community composition) (2), or which genes are present in the community (15), but rather which genes are expressed and in which genomic background; in other words, the key is the breadths of the Hutchinson ecological niches of organisms encoding the biodegradation function in their genomes (Fig. 2). Indeed, the persistence of a given function will depend on the capacity of the gene, pathway, and/or host organism of interest to survive perturbation and to the ability of resistance/resilience capacity of the community as a whole. The rapid expansion of omics is providing us with mature technologies (7) to tackle such crucial issues in a renewed way. Thus, the time is ripe to produce new knowledge which will be directly transferable to modeling, diagnostics, and environmental biotechnology applications. In the more distant future, moving beyond basic ecological classifications of lifestyle strategies for microbes such as generalists and specialists, towards more specific classifications such as the universal adaptive strategy theory (UAST), which describes tradeoffs between ruderal, stress-tolerant, and competitor behaviors (16, 17), might even further improve the predictability of our models.
